# Totally endoscopic trans-mitral removal of a left ventricular apical thrombus through right thoracotomy in a patient with subacute anterior myocardial infarction: a case report

**DOI:** 10.1186/s44215-023-00055-0

**Published:** 2023-07-18

**Authors:** Ryoma Oda, Atsumi Oishi, Yuki Kamikawa, Hiroaki Hata, Kan Kajimoto

**Affiliations:** Department of Cardiovascular Surgery, Juntendo Shizuoka Hospital, Nagaoka 1129, Izunokuni, Shizuoka 410-2211 Japan

**Keywords:** Endoscopic thrombectomy, Apical thrombus, Minimally invasive surgery, Subacute myocardial infarction

## Abstract

**Background:**

Left ventricular thrombosis confers a life-threatening risk of systemic embolism; therefore, it requires prompt intervention. Although anticoagulation is the primary treatment, surgery is indicated in instances of large or/and mobile thrombus or when there is potential for recovery of ventricular contraction. However, standard left ventriculoplasty with thrombectomy carries risks of cardiac dysfunction due to left ventriculotomy.

**Case presentation:**

A 70-year-old man developed chest pain and vomiting 3 weeks before presenting to our hospital. A chest radiograph showed substantial cardiomegaly and mild pulmonary congestion; N-terminal pro-brain natriuretic peptide (5698 ng/L) was substantially increased, and troponin T (56 ng/L) levels were slightly above reference values. Transthoracic echocardiography showed akinesis of the anteroseptal and apical segments with an ejection fraction reduced to approximately 20%. We diagnosed subacute myocardial infarction and initiated pharmacotherapy. On hospital day 7, coronary angiography revealed a left anterior descending artery lesion with 99% stenosis; percutaneous coronary intervention was successfully performed the next day. That same day, transthoracic echocardiography revealed a large mobile left ventricular apical thrombus without any left ventricular aneurysm, and heparin therapy was initiated. On hospital day 10, three-dimensional computed tomography confirmed the location of an apical thrombus, and we planned a fourth intercostal approach. A thrombectomy was performed on hospital day 11 using an endoscopic trans-mitral approach with a right thoracotomy to avoid a left ventriculotomy. The patient was discharged from intensive care on postoperative day 2 under heparin and warfarin therapy. The subsequent postoperative course was uneventful, and he was discharged on postoperative day 14 with a vitamin K antagonist. At the 6-month follow-up, there was no recurrence of thrombus in the left ventricle and Ejection Fraction had improved to 46%.

**Conclusions:**

Totally endoscopic thrombectomy via a trans-mitral approach through right thoracotomy was effective for a left ventricular thrombus. When concomitant coronary artery bypass grafting or left ventriculoplasty are not required, this procedure can be an effective option.

## Background

Left ventricular thrombosis occurs in patients with left ventricular hypocontractility associated with acute myocardial infarction [1]. Left ventricular thrombosis carries a life-threatening risk of systemic embolism and requires prompt intervention. Although anticoagulation is the primary treatment, surgery is indicated in patients with large and/or mobile thrombi or the potential for recovery of ventricular contraction [2]. Left ventriculoplasty with thrombectomy is the standard surgical procedure. However, transient left ventricular hypocontractility associated with ventriculotomy often prolongs intensive care unit (ICU) and hospital stays. Herein, we describe a totally endoscopic thrombectomy without ventriculotomy in a patient with a massive apical thrombus associated with subacute myocardial infarction.

## Case presentation

A 70-year-old man developed chest pain and vomiting 3 weeks before presenting to our hospital. Later, he developed tightness of chest and dyspnea on light exertion. At our hospital, a chest radiograph showed substantial cardiomegaly and mild pulmonary congestion. Blood tests revealed a creatine kinase level within the reference range, while levels of *N*-terminal pro-brain natriuretic peptide (5698 ng/L, reference range 0–55) and troponin T (56 ng/L, reference range 0–14) were increased. Electrocardiography showed a QS-pattern in V1–V4 and no ST-segment elevation. Transthoracic echocardiography revealed akinesis of the anteroseptal and apical segments with an ejection fraction reduced to approximately 20%. Subacute myocardial infarction was diagnosed; pharmacotherapy was initiated since the patient had no chest pain and was in congestive heart failure with demand for oxygen. Coronary angiography was implemented promptly after the initial management of heart failure.

**Fig. 1:**
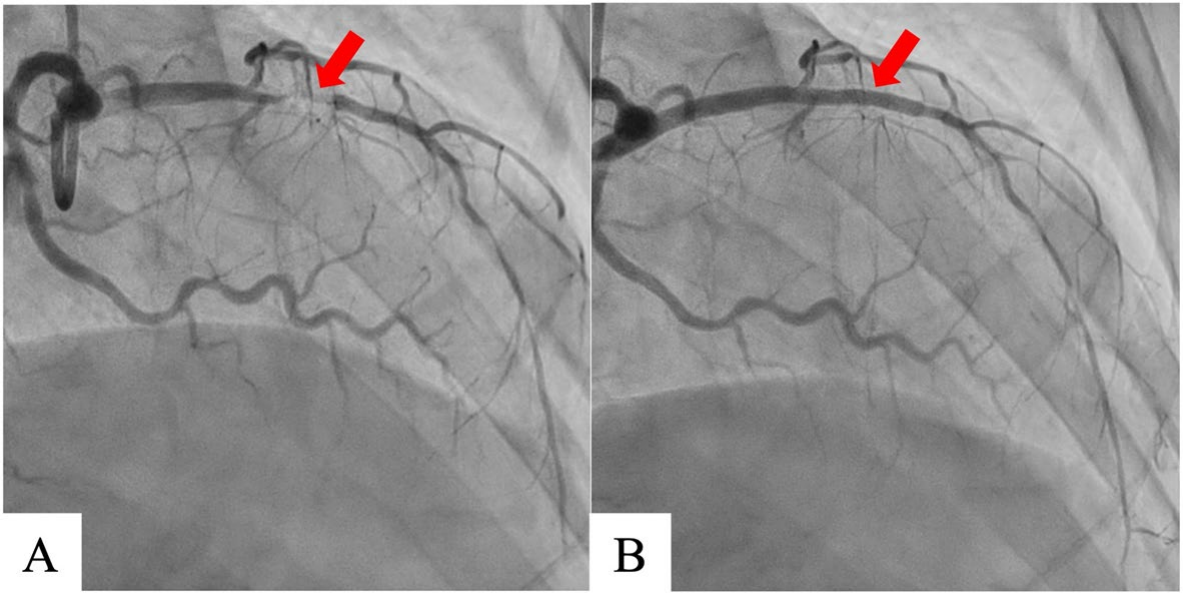
Coronary angiography shows left anterior descending artery with 99% stenosis (#7) (**A**). Percutaneous coronary intervention was performed successfully (Ultimaster Tansei 3.0 × 24 mm, TERUMO, Japan) (**B**)

**Fig. 2:**
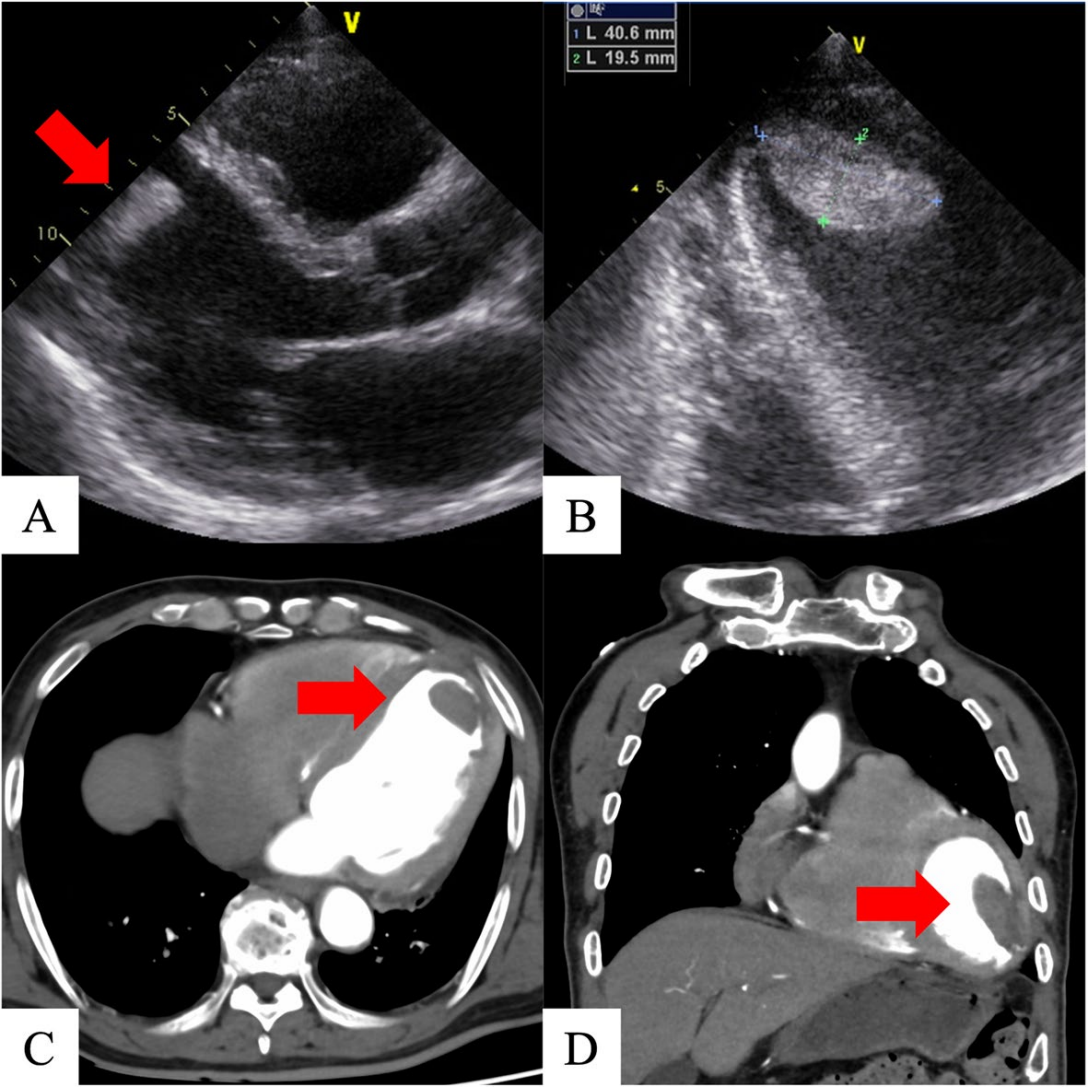
Preoperative transthoracic echocardiography and computed tomography. Transthoracic echocardiography shows anteroseptal and apical wall motion with akinesis and an apical thrombus (**A**). Transthoracic echocardiography shows a massive apical thrombus (40.6 × 19.5 mm) (**B**). Three-dimensional computerized tomography also shows the apical thrombus (**C**, **D**)

On hospital day 7, coronary angiography revealed a left anterior descending artery (#7) lesion with 99% stenosis; percutaneous coronary intervention was successfully performed the next day (Fig. 1). The same day, transthoracic echocardiography revealed a large mobile left ventricular apical thrombus without a left ventricular aneurysm, not present at the time of admission (Fig. 2A, B), and heparin therapy (target control activated partial thromboplastin time, 1.5–2.0 times the normal range) was initiated. On hospital day 11, considering the thrombus size and mobility, the prospect of improved cardiac contractility, and no significant improvement with anticoagulation, surgery was performed. An endoscopic trans-mitral approach through minimally invasive right thoracotomy was selected to avoid a left ventriculotomy. Three-dimensional computed tomography (3D-CT) performed the day before surgery confirmed the location of the apical thrombus (Fig. 2C, D); hence, we planned a fourth intercostal approach.

**Fig. 3:**
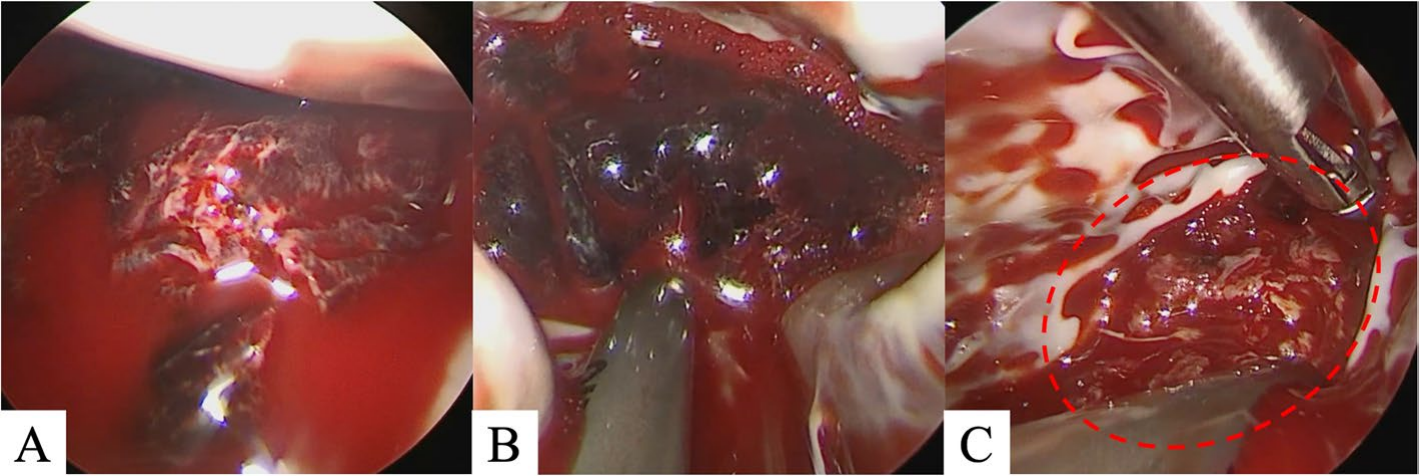
Intraoperative findings: left ventricular thrombus visualized using an endoscope. Before thrombectomy: A large black thrombus is noted in the apical left ventricular region (**A**). During thrombectomy, thrombectomy was performed with a suction device (**B**). After thrombectomy, the black thrombus is removed; however, the white thrombus is firmly attached to the left ventricular wall (**C**)

**Fig. 4:**
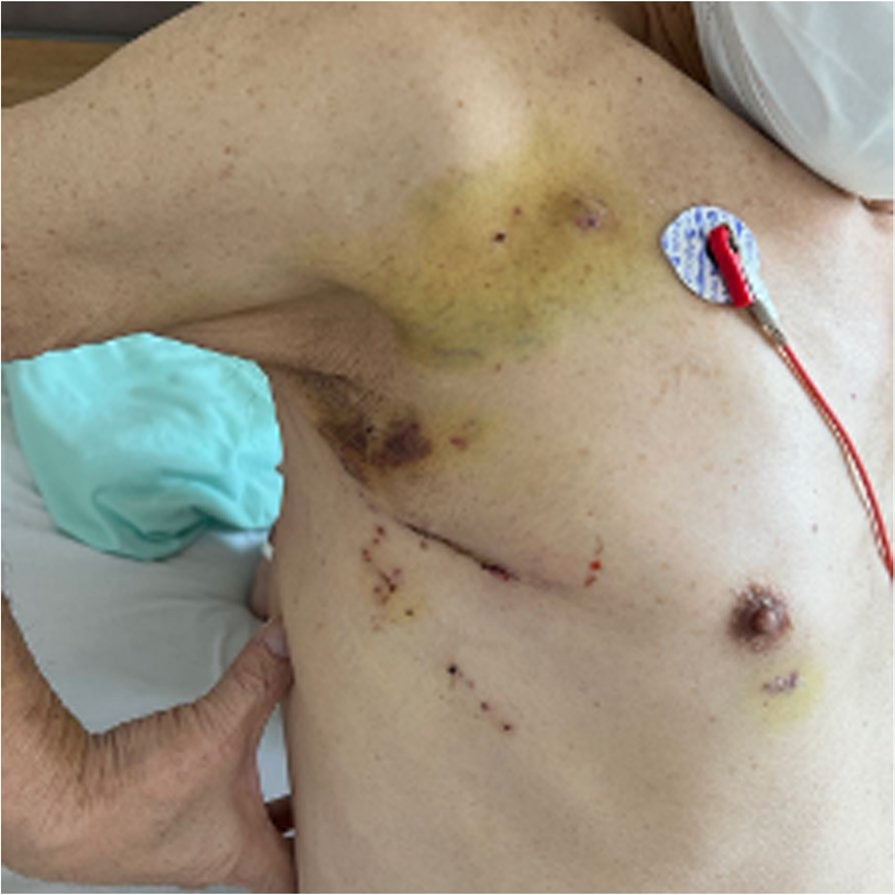
Postoperative wound on the day of discharge

Under general anesthesia, the patient was placed in a supine position with the right side elevated at approximately 20°. An approximately 4.5 cm skin incision was made in the right fourth intercostal space, and a skin incision of approximately 2.5 cm was made in the right inguinal region. Cardiopulmonary bypass was established from the superior vena cava (internal jugular vein access) and inferior vena cava (femoral vein access) to the right femoral artery. The camera port was placed at the fifth intercostal anterior axillary line, and a 10-mm 30° rigid endoscope (ENDOSKOPE, KARL STORZ SE & Co. KG, Tuttlingen, German) was placed. An aortic cross-clamp (ValveGate™ Transthoracic Aortic Clamp Geister Medizintechnik GmbH, Tuttlingen, Germany) was inserted from the right second intercostal space, and the aorta was clamped under camera guidance. Antegrade cardioplegia was delivered into the aortic root. After cardiac arrest, a right-sided left atrial incision was made. An HV Heart retractor (USB Medical, Huntingdon Valley, PA) was placed through a 5-mm incision at the right margin of the third right intercostal sternum. The mitral leaflets were retracted using the Wakka technique [3], and the apical thrombus was observed trans-mitrally (Fig. 3A). Thrombectomy was performed while avoiding damage to the subvalvular mitral tissue. The black thrombus was very fragile; we performed suction and saline irrigation using “HiQ + suction and water pipes” (OLYMPUS, Tokyo), usually used for laparoscopic surgery (Fig. 3B). Using this device, we were able to remove the jelly-like part of the thrombus by direct suction under negative pressure. Furthermore, the remaining part was excised with rongeur forceps and suctioned while fracturing it with forceps without dispersing the fragments. Finally, the left ventricle cavity was thoroughly rinsed and aspirated with saline to ensure no residual black clots were present. Although the white thrombus that was firmly adherent to the left ventricle could not be completely removed, the black thrombus was thoroughly removed, minimizing the risk of embolization (Fig. 3C). Cardiopulmonary bypass was easily weaned off without arrhythmia. Intraoperative transesophageal echocardiography displayed no evidence of any residual thrombus. Postoperatively, the patient was transferred to the ICU and weaned from the ventilator 10 h later. On postoperative day 2, he was discharged from the ICU under heparin and warfarin therapy. Postoperative echocardiography displayed no residual left ventricular thrombi, and the left ventricular ejection fraction had improved to 39%. 3D-CT displayed no recurrent left ventricular thrombi. The patient’s postoperative course was uneventful, and he was discharged on postoperative day 14 with a vitamin K antagonist (Fig. 4). At the 6-month follow-up, there was no recurrence of thrombus in the left ventricle and Ejection Fraction had improved to 46%.

## Discussion

A left ventricular thrombus is associated with left ventricular hypocontractility and can be caused by acute myocardial infarction, idiopathic dilated cardiomyopathy, myocarditis, critical valvular disease, or severe ischemic cardiomyopathy. It occurs in at least 5% of acute myocardial infarctions [1]. Although anticoagulation is the primary treatment, surgery has been conventionally indicated in patients with large and/or mobile thrombi or the potential for recovery of ventricular contraction [2]. This patient fulfilled all the three criteria; the thrombi were huge and significantly mobile. Therefore, given the risk of systemic embolism, we considered the patient for surgery. In contrast, the American Heart Association (AHA) issued a scientific statement regarding “Management of Patients at Risk for and With Left Ventricular Thrombus” in October 2022 [4]. In this statement, the AHA recommended anticoagulation therapy and concluded that the currently available data are insufficient to recommend surgical excision. Therefore, careful consideration is needed regarding the indications for surgery in each case.

The surgery itself may pose a risk of embolism, and residual thrombus is possible [5]. Therefore, a safe and reliable procedure is necessary. Conventionally, thrombectomy is performed by a trans-left ventricular approach under direct vision; however, left ventricular dysfunction caused by ventriculotomy sometimes leads to low cardiac output syndrome. When left ventricular thrombosis is accompanied by a left ventricular aneurysm, left ventriculoplasty is performed in addition to thrombectomy. A systematic review reported 9.3% of patients undergoing left ventriculoplasty exhibit low output syndrome and 18.8% required an intra-aortic balloon pump [6]. Even in the absence of a left ventricular aneurysm, it is difficult to clearly delineate the boundary between normal and infarcted myocardium, and it is impossible to perform a left ventriculotomy without damaging the normal myocardium at all. Because of this, other approaches have been attempted in recent years.

Williamson et al. reported a trans-aortic, video-assisted removal of a mobile left ventricular apical thrombus in a patient with aortic stenosis and severe left ventricular dysfunction [7]. Moreover, endoscopic thrombectomy or endoscopic-assisted thrombectomy via a trans-mitral approach has also been reported in recent years [5, 8, 9]. These two approaches, namely the trans-aortic valve and left atrial trans-mitral, achieved good surgical outcomes by avoiding left ventriculotomy.

In our case, the left ventricular thrombus without a left ventricular aneurysm was detected after percutaneous coronary intervention. Therefore, we performed left ventricular thrombus removal without revascularization or left ventriculoplasty. The trans-mitral approach with minimally invasive right-sided thoracotomy minimizes postoperative cardiac dysfunction by avoiding ventriculotomy. The use of an endoscope improves the visibility of the left ventricular cavity and allows thrombectomy without damaging subvalvular tissue. Furthermore, the Wakka technique, as reported for endoscopic mitral valve surgery, ensures a clearer vision of the left ventricular cavity. The concern that the fragile thrombus could easily collapse was addressed using the HiQ + suction system, which enabled us to aspirate clots without disseminating or losing fragments. Endoscopic thrombectomy for patients with reduced left ventricular function, as in this patient, has been reported [8]. Although there are no guidelines regarding recommendations or contraindications for minimally invasive cardiac surgery for left ventricular dysfunction, care is needed when cardiopulmonary bypass is weaned off.

Totally endoscopic trans-mitral left ventricular apical thrombectomy through minimally invasive right-sided thoracotomy was an effective approach in treating our patient. and should be considered in cases of intraventricular thrombus complicating myocardial infarction. When concomitant coronary artery bypass grafting or left ventriculoplasty are not required, this procedure can be an effective option. Its advantages include minimal invasiveness, reliable thrombectomy, and avoidance of low cardiac output syndrome caused by left ventriculotomy.

## Data Availability

Data sharing is not applicable to this article, as no datasets were generated or analyzed during the current study.

## Abbreviations

AHA: American Heart Association
ICU: Intensive care unit
3D-CT: Three-dimensional computed tomography
